# Supplementary material for: Central transcriptional regulator controls photosynthetic growth and carbon storage in response to high light
Source: Nat Commun. 2024 Jun 6;15:4842. doi: 10.1038/s41467-024-49090-7 (PMC11156908; doi:10.1038/s41467-024-49090-7)
Supplement: Supplementary file 1 — Supplementary information [file 41467_2024_49090_MOESM1_ESM.pdf]

Central Transcriptional Regulator Controls Photosynthetic Growth and Carbon Storage in Response to High Light

Steichen, S.[1], Deshpande, A. [1], Mosey, M. [1], Loob, J. [1], Douchi, D.[1], Knoshaug, E.P.[1], Brown, S.[2], Nielsen, R. [2], Weissman, J. [2], Carrillo, L.R. [2], Laurens, L.M.L.* [1]

[1] Bioenergy Science and Technology Directorate, National Renewable Energy Laboratory, 15013 Denver West Parkway, Golden, CO 80401

[2] ExxonMobil Technology and Engineering Co. (EMTEC); 1545 Route 22 East, CLD286 Annandale, NJ 08801

*author for correspondence (lieve.laurens@nrel.gov)

Supporting Information for Instationary Metabolic Flux Analysis (INST-MFA)

It is of great value to study metabolism with minimal perturbation of the system to accurately represent metabolism. ^{13}C stable isotope labeling allows investigation of cellular metabolism *in-vivo* in comparison to other *in-vitro* approaches such as enzyme kinetics. In addition, fluxomics represents the end result of the interplay between the genome, transcriptome, proteome, metabolome, and epigenome and is the ultimate representation of the phenotype. This document reports all supplementary figures and details related to characterization of metabolic flux.

Supporting Methods:

HILIC LC-MS Method: The metabolites were separated using hydrophilic interaction chromatography (HILIC) using a BEH Amide column (1.7 µm, 2.1 mm X 150 mm, ACQUITY Premier BEH Amide, Waters Corporation) via a gradient method. Solvent A comprised of 20 mM ammonium acetate and 15 mM ammonium hydroxide in 97% 18.2 mΩ-cm water and 3% acetonitrile whereas solvent B comprised of 20 mM ammonium acetate and 15 mM ammonium hydroxide in 95% acetonitrile and 5% 18.2 mΩ-cm water. All the chemicals used were LC-MS grade. The chromatography method used a constant flow rate of 0.2 mL/min and a linear gradient to enable separation of a wide range of metabolites followed by a column regeneration step as follows: 90% solvent B for 1 min followed by a linear gradient down to 75% solvent B for 23 min, a linear gradient down to 45% solvent B in 2 min, a linear gradient down to 25% B in 4 min, followed by a step to the starting composition of 90% B and hold for 6 min for column regeneration for a total run time of 36 min. The column temperature was maintained at 25°C and injection volume was set to 20 µL. Metabolite extracts were diluted such that the final injection solvent was 75% acetonitrile. Data was collected using a Thermo Scientific Q-Exactive orbitrap mass spectrometer using electrospray ionization (ESI) in negative ion mode. The sheath gas, aux gas, and sweep gas flow rates were 45, 8, and 1 a.u. respectively. Spray voltage was 3.5 kV and capillary temperature was 320°C. S-lens RF level was 50. Data were collected using full scan mode with scan range from 75 to 1000 m/z with a resolution of 140,000. AGC target was set to 3e6 while maximum IT was 200 ms. The retention times of some of the targeted metabolites analyzed are listed in

Supplementary Table 10. Metabolites were identified using authentic standards and matching retention times. Data was integrated using Tracefinder 5.1 (Thermo Fisher Scientific) and analyzed using R (4.2.0).

Three strains, each with three biological replicates (n = 3), and 6 timepoints were analyzed (54 samples). Data was collected in 9 separate runs, one for each strain and replicate. For each run, blank (75% ACN) and quality control (pooled sample consisting of equal volumes of all samples in the batch) samples run. Samples were run in a randomized order.

Amino Acid Composition of Protein: The total amino acid composition of the samples was determined after hydrolysis at 110°C for 24h with 6N HCl. An aliquot of the amino acid hydrolysate was taken and diluted 100-fold in 75% acetonitrile. Samples were analyzed for amino acids using the HILIC LC-MS method as described above.

A preliminary experiment was performed to test the method and compare with quality control samples of different algae strains with commercial vendor aminoacids.com as well as the analysis via o-phthalaldehyde (OPA) and 9-fluorenylmethyl chloroformate (FOMC) derivatization method followed by separation and detection using Zorbax Eclipse-AAA column and diode array detector (DAD) detector. Glutamic acid and glutamine are reported as 0.5 times the measured glutamic acid content since sample preparation results in all the glutamine to convert to glutamic acid. Tryptophan is degraded in the sample preparation and thus is assumed to be that reported by aminoacids.com for TG2.The amino acid data for method testing is shown in **Supplementary Fig 17**. while **Supplementary Fig 16**. shows the amino acid composition of TG1, TG2, and TG1-MYB99 under different cultivation conditions. The data represents the mean ± SD of three biological replicates (n = 3). **Supplementary Table 6.** also shows the measured data used in the development of the protein synthesis equation for the INCA model.

Chlorophyll Content Regression

The concentration of chlorophyll in cell suspension samples was calculated using a linear regression based on optical absorbance measurements. The background absorbance was calculated using a linear curve between two non-absorbing regions at 750 nm and 565 nm, which was subtracted from the chlorophyll absorbing peak at 682 nm. The overall linear regression was established for TG1 and TG2 samples separately by determining a slope coefficient (β) based on comparison to measured chlorophyll content determined by an ethanol extraction method[1] using the equation below. The β values utilized were 25.66 and 22.66 for TG1 and TG2 samples, respectively.

$$Total\ CHL\ \left(\frac{\mu g}{mL}\right) = \left(OD682 - \left(\left(\frac{OD750 - OD565}{185}\right) * 682 + \left(OD750 - \left(\frac{OD750 - OD565}{185}\right) * 750\right)\right)\right) * \beta$$

INST-MFA Assumptions:

1. A significant advantage of INCA is that pool sizes can be treated as parameters to be estimated and do not have to be measured quantitatively. Our model treats pool sizes as free parameters

that are estimated. This approach has been utilized previously in flux studies in cyanobacteria as well as algae[2,3].

2. Although a complete and instantaneous shift from ^{12}C to ^{13}C is desirable, it may not be possible to achieve experimentally. Bolus addition of $NaH^{13}CO_3$ at 11.76 mM is able to achieve result in an instantaneous and almost complete switch to ^{13}C uptake, however there is likely to be some residual ^{12}C. Previous work in flux work cyanobacteria showed that the best fit was obtained under 97% ^{13}C as a model input[2]. We therefore performed simulations considering that 97% of the CO_2 fixed was labelled.
3. INST-MFA relies on the assumption that metabolic fluxes and pool sizes do not change throughout the course of the labeling experiment and are not perturbed by addition of labeled $NaH^{13}CO_3$.
4. The algal strains do not discriminate between labelled and unlabeled carbon during photosynthesis.
5. Several assumptions were made for developing the biomass composition reactions. For nucleotides, DNA and RNA biomass fraction as well as the AT/UGC composition was assumed to be that measured in *Chlorella protothecoides*[4]. This resulted in one mole of DNA and RNA measuring 339.86 g/mol and 340.41 g/mol respectively. The chlorophyll content was assumed to be that measured under high light in *Picochlorum celeri*[1] with a molecular weight of 893.51 g/mol. One unit of carbohydrate weighed 260.14 g/mol (since it is modeled as G1P + ATP -> Carbohydrates) whereas a lipid model was based on C18 Fatty acid and weighed 284.49 g/mol. As each strain had a measured distribution of amino acids in protein, the mass of an amino acid unit was modeled as 128.12 g/mol for TG1, 128.07 g/mol for TG2, and 128.36 g/mol for TG1-MYB99.
6. Experimentally determined biomass composition often does not satisfy mass balance due to factors such as ash content, underestimation of components etc. Therefore, it is important to address this to adequately represent the biomass composition input to the INCA model. To do this, experimental data was first appended to include assumed values for DNA, RNA and Chlorophyll content. This was followed by scaling FAME by a factor of 1.5x since these are often underestimated. Next, carbohydrate content was scaled by a factor of 1.1x for high carbohydrate species and 1.3x for low carbohydrate species. Since samples with a high carbohydrate content consist largely of glucose which is easier to release, a different factor was chosen compared to low carbohydrate samples. Next, the resulting composition was expressed on an ash free basis and finally scaled such that the sum of all components satisfied mass balance. The biomass composition and specific growth rate used for the INCA model biomass equation formulation and sink demand is shown in **Supplementary Table 4 and 5** respectively.
7. Individual biomass equations were developed for carbohydrate, nucleotide, protein, lipid, and chlorophyll biosynthesis for each strain. The equations used in the INCA model was formulated based on the reactions listed in **Supplementary Table 7.** to calculate precursor demands for metabolites that are part of the INCA model. Together with the specific growth rates in **Supplementary Table 5.**, a sink demand was calculated and given as an input to the models.

Supplemental Tables and Figures

Supplemental Tables

Supplementary Table 1. TG1 variant sites functional context

Context	SAMtools all	SAMtools filter	GATK filtered
downstream	17,978	17,690	8,806
exonic	226,035	224,868	135,822
exonic;splicing	1	1	3
intergenic	16,548	15,697	2,281
intronic	36,834	36,202	20,860
splicing	786	781	486
upstream	45,159	44,738	26,987
up;downstream	41,553	41,232	26,617
UTR3	560	524	235
UTR5	1,913	1,872	832
UTR5;UTR3	4	NA	NA
total	387,371	383,605	222,929

Supplementary Table 2. Light-Responsive transcriptome comparisons

Species	Time in high light (min)	Light (µE)	p-value cutoff[††]	L2FC cutoff	Proportion of transcripts changed (%)	Reference
C. reinhardtii	40	41 -> 145*	0.05**	0	15	Mettler, 2014
P. celeri TG1	60	60 -> 1000	0.05[†]	0	72	Current Study
P. celeri TG2	60	60 -> 1000	0.05[†]	0	73	Current Study
C. reinhardtii	40	41 -> 145*	0.05**	3	6	Mettler, 2014
P. celeri TG1	60	60 -> 1000	0.05[†]	3	8	Current Study
P. celeri TG2	60	60 -> 1000	0.05[†]	3	6	Current Study
C. zofingiensis	60	100 -> 400*	0.01	2	3	Roth, 2017
P. celeri TG1	60	60 -> 1000	0.01[†]	2	13	Current Study
P. celeri TG2	60	60 -> 1000	0.01[†]	2	11	Current Study
A. thaliana	30	100 -> 1000	0.05	3	3	Crisp, 2017
A. thaliana	1	50 -> 1000	0.05	2	1	Suzuki, 2015

*not saturating

** Calculated by ANOVA contrast

[†]P-values determined by two-sided Wald test followed by Benjamini-Hochberg multiple testing adjustment.

[††]Readers are referred to individual references for specific details on statistical analyses methodologies.

Supplementary Table 3. TG1-MYB99 transgene insertion site overhang BLASTn alignment loci

query acc.ver	subject acc.ver	% identity	alignment length	mismatches	gap opens	q. start	q. end	s. start	s. end	e-value	bit score
NODE_1_length_1537_cov_81.771631	EMRE3EUKT4066622	99.455	550	3	0	988	1537	1	550	0	1000
NODE_1_length_1537_cov_81.771631	EMRE3EUKT4066598	99.455	550	3	0	988	1537	1	550	0	1000
NODE_1_length_1537_cov_81.771631	EMRE3EUKT4063967	99.273	550	4	0	988	1537	1	550	0	994
NODE_1_length_1537_cov_81.771631	EMRE3EUKT4068533	98.727	550	7	0	988	1537	1	550	0	977
NODE_1_length_1537_cov_81.771631	EMRE3EUKT4067234	98.364	550	9	0	988	1537	1	550	0	966
NODE_1_length_1537_cov_81.771631	EMRE3EUKT4068995	95.229	545	26	0	988	1532	1	545	0	863
NODE_1_length_1537_cov_81.771631	EMRE3EUKT4067887	98.276	58	1	0	1480	1537	1	58	1.79E-21	102

Supplementary Table 4. Biomass composition (%) used to formulate biomass component synthesis equation for the INCA model. DNA and RNA content was assumed from *Chlorella protothecoides*[4] while chlorophyll content was assumed from *P. celeri*[1] and scaled to satisfy mass balance. Data are reported as the mean ± SD from three biological replicates (n = 3). Biomass was sampled from cultures grown under the same conditions on which the isotopic labeling experiment was performed. * indicates insufficient biomass for remaining for analysis and is reported as only one replicate.

Strain	FAME (%)	Protein (%)	Carbs (%)	DNA (%)	RNA (%)	Chl A (%)
TG1	11.84 ± 0.21	29.9 ± 0.09	44.86 ± 0.27	3.43	7.66	2.31
TG2	16.93 ± 0.27	50.84*	16 ± 1.07	3.86	8.62	2.6
TG1-MYB99	9.46 ± 0.29	21.03 ± 2.98	56.14 ± 2.68	3.42	7.64	2.3

Supplementary Table 5. Specific growth rate of three strains used in flux analysis for calculation of sink demand. Data represents mean ± SD of three biological replicates (n = 3) used in the flux analysis.

Strain	Specific growth rate (h^{-1})[a]
TG1	0.175±0.024
TG2	0.239±0.016
TG1-MYB99	0.182±0.048

[a] Specific growth rate shown is based on estimated chlorophyll from optical density measurements. Under low density and high light irradiation conditions, all the three strains show the same chlorophyll content which enables growth rate calculation based on chlorophyll estimation.

Supplementary Table 6. Measured amino acid composition of hydrolyzed protein of strains grown under high light conditions. The data represents mean ± SD of three biological replicates (n = 3). † indicates 0.5 times the measured aspartic acid content since sample preparation results in all the asparagine to convert to aspartic acid. †† indicates 0.5 times the measured glutamic acid content since sample preparation results in all the glutamine to convert to glutamic acid. In both these cases, these simplifying assumptions were made in order to write the protein synthesis equation. ** indicates amino acid is degraded in the sample preparation and thus is assumed to be that reported by aminoacids.com for TG2 (cultivated at HL to a high cell density). * indicates that amino acid did not have an a reliable peak and was assumed to be % reported by aminoacids.com for TG2.

**Amino Acid**	**TG1 (%)**	**TG1-MYB99 (%)**	**TG2 (%)**
Alanine	12.09±0.23	11.85±0.06	12.45±0.2
Arginine	7.99±0.21	8.83±0.5	7.67±1.33
Aspartic acid	4.69±0.09†	4.66±0.21†	4.67±0.34†
Asparagine	4.69±0.09†	4.66±0.21†	4.67±0.34†
Cysteine	0.53±0.06	0.48±0.06	0.65±0.05
Glutamic acid	7.44±0.13††	7.19±0.35††	6.76±0.2††
Glutamine	7.44±0.13††	7.19±0.35††	6.76±0.2††
Histidine	1.9±0.15	1.76±0.31	3.05±0.44
Isoleucine	2.6±0.38	3.13±0.21	3.15±0.1
Leucine	5.21±0.76	6.26±0.42	6.3±0.2
Lysine	6.26±0.29	6.09±0.23	5.45±0.82
Methionine	0.82±0.02	0.76±0.2	0.87±0.13
Serine	5.49±0.13	5.26±0.21	4.82±0.23
Threonine	3.31±0.18	3±0.17	3.05±0.19
Tyrosine	2.34±0.35	1.94±0.25	2.53±0.13
Valine	7.19±0.35	6.94±0.28	7.16±0.38
Phenylalanine	4*	4*	4*
Tryptophan	1.2**	1.2**	1.2**
Glycine	8.8*	8.8*	8.8*
Proline	6*	6*	6*

Supplementary Table 7. Equations used to develop biomass synthesis equations. These equations were used to determine the precursors required to produce protein, nucleotides, lipids, chlorophyll, and carbohydrates. The stoichiometric molar quantity for precursor demand was quantified per kg DCW.

**Carbohydrate**[5]
G1P + ATP -> Carbohydrates
**Chlorophyll A**[2]
8 Glu+4 GAP+4 PYR -> Chl A
**Nucleotides**[4]
R5P + 2 ASP + GLY + 2 GLN + 7 ATP + 2FTHF + HCO3 - -> AMP + 2 FUM + 2 GLU
R5P + ASP + GLY + 3 GLN + 7 ATP + 2 FTHF + HCO3 -> GMP + FUM + NADH + 3GLU
PRPP + GLN + 2 ATP + ASP -> UMP + GLU + NADH
PRPP + 2 GLN + 3 ATP + ASP -> CMP + 2 GLU + NADH
UMP + MTHF -> TMP + THF
**Lipids**[5]
9 AcCoA + 16 NADPH + 8 ATP-> C18 FATTY ACID + 9 CoA + 8 ADP + 16 NADP+
**Protein (Amino Acids)**[4,5]
PYR + GLU -> ALA + AKG
2 GLU + AcCoA + CP + ASP + 2 ATP + NADPH -> ARG + AKG + FUM + Ac + CoA + 2 ADP + NADP+
NH3 + ASP + ATP -> ASN + ADP
OAA + GLU -> ASP + AKG
SER + AcCoA + H2S -> CYS + Ac + CoA
NH3 + GLU + ATP -> GLN + ADP
AKG + NH3 + NADPH -> GLU + NADP+
SER + THF -> Gly + 5,10 Me-THFA
R5P + PRPP + GLN -> HIS
THR + NH3 + PYR + GLU + 6 NADPH -> ILE + AKG + CO2
2 PYR + AcCoA + GLU -> 2 CO2 + AKG + LEU + CoA
ASP + SUCCoA + GLU + PYR + 2 NADPH + ATP -> LYS + AKG + CoA + CO2 + Suc
ASP + SUCCoA + CYS + 5MeTHF + ATP + 2 NADPH -> MET + SUC + CoA + PYR + NH3 + THF + ADP + 2 NADP+
E4P + 2PEP + ATP + NADPH + GLU -> PHE + AKG + CO2 + ADP
GLU + ATP + 2 NADPH -> PRO + NADP+ + ADP
3PGA + GLU + NAD+ -> SER + AKG + NADH
ASP + 2 ATP + 2 NADPH -> THR
E4P + 2PEP + ATP + NADPH + PRPP + GLN + SER -> TRP + G3P + GLU + CO2 + ADP
E4P + 2PEP + ATP + NADPH + GLU + NAD+ -> TYR + AKG + CO2 + NADH + NADH
2 PYR + GLU -> VAL + CO2 + AKG

Supplementary Table 8. Photophysiology FRRf metrics

Strain	Light Acclimation	Fv/Fm	470 Sigma (nm^2)	535 Sigma (nm^2)	p	Tau1QA (µs)	Tau2QA (µs)	Tau3QA (µs)
**TG1-MYB99**	HL	0.6 ± 0.04	1.24 ± 0.05	0.61 ± 0.01	0.28 ± 0.24	1154.33 ± 905.6	382 ± 103.36	5781.67 ± 3897.81
**TG1-WT**	HL	0.59 ± 0.05	1.34 ± 0.05	0.58 ± 0.05	0.3 ± 0.19	620.67 ± 17.9	421.33 ± 59.47	5148 ± 301.38
**TG2-WT**	HL	0.57 ± 0.01	1.11 ± 0.13	0.51 ± 0.03	0.16 ± 0.18	1005 ± 271.93	269 ± 8.66	3923.33 ± 1043.27
**UTEX393***	LL	0.3558	1.7912	4.1182	0.26	1092	857	28209

*UTEX393 is a strain of *Scenedesmus obliquus*. A single replicate measurement of UTEX393 cell culture growing under 60 µE PAR was collected to provide a point of comparison for the FRRf as implemented during this study.

**Photosynthetic parameters were determined by fitting a three-component differential equation model to single turnover flash transients as described previously[6,7]

***Variable fluorescence (Fv/Fm) determined on classical, unbounded, scale. The 470 Sigma and 535 Sigma values represent functional antenna cross sections based on 470 nm and 535 nm single turnover flash responses, respectively. The p parameter defines the probability of excitation energy exchange between individual reaction centers. Each TauQA parameter represents a time constant for sequential electron transfer steps based on relaxation kinetics following a saturating single turnover flash.

Supplementary Table 9. Primer sequences used to amplify MYB1 locus (promoter – CDS – terminator) and confirm insertion of pGAPDHnat-MYB1 in the genome. Lower case letters denote overlap with vector for in-fusion cloning purposes.

Primer name	Sequence (5' to 3')
**MM01**	tcatagtgtacatggagtcgactagctcgagTGGGATGAGCAAGGTGCTGC
**MM02**	agtcgaaagactgggcctttcgcccgagctcTACGGTCAATCAGTTCGAAACGTGG
**MM03**	CCCAGATACATCTCAGGCTGTCAT
**DD235**	TGCTGAGGCTATTGAGGCTTTG

Supplementary Table 10. Selected metabolite retention times using HILIC chromatography method

Metabolite	Retention time (min)
Pyruvate (PYR)	4.75
Lactic Acid	5.92
Alanine (ALA)	7.35
Alpha-ketoglutarate (AKG)	8.38
Succinate (SUC)	8.46
Malate (MAL)	9.73
Fumarate (FUM)	9.73
Glyceraldehyde-3-phosphate (G3P)	10.28
Ribose-5-phosphate (R5P)	10.35
Fructose-1,6-bisphosphate (FBP)	10.95
Dihydroxyacetone phosphate (DHAP)	10.95
Erythrose-4-phosphate (E4P)	11.28
Fructose-6-phosphate (F6P)	11.29
2-phosphoglycerate (2PGA)	11.67
Sedoheptulose-7-phosphate (S7P)	11.82
Phosphoenolpyruvate (PEP)	11.85
3-phosphoglycerate (3PGA)	11.97
Citrate/Isocitrate (CIT)	12.31
Glucose-6-phosphate (G6P)	12.37
Oxaloacetate (OAA)	12.4

Supplemental Figures

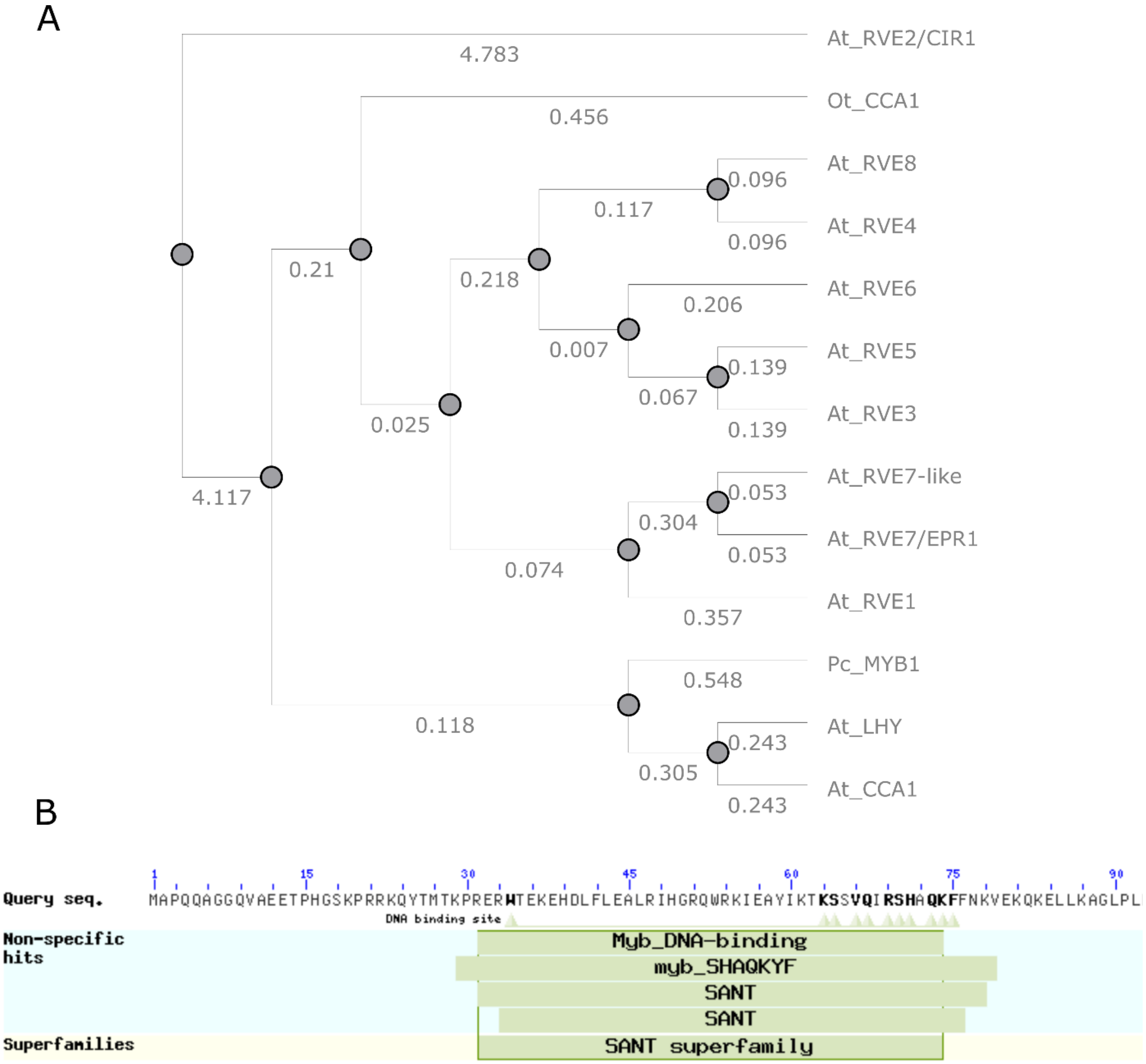

Supplementary Figure 1. *Picochlorum celeri* CCA1/LHY protein sequence analysis. Phylogenetic tree (A) displaying the relationship between the *P. celeri* CCA1/LHY (Pc_MYB1) homologous protein sequence and related *A. thaliana* gene product sequences. Conserved domain (B) locus identified within the first 80 amino acid residues of the Pc_MYB1 sequence. The remaining sequence results were truncated as there were no significant matches identified therein.

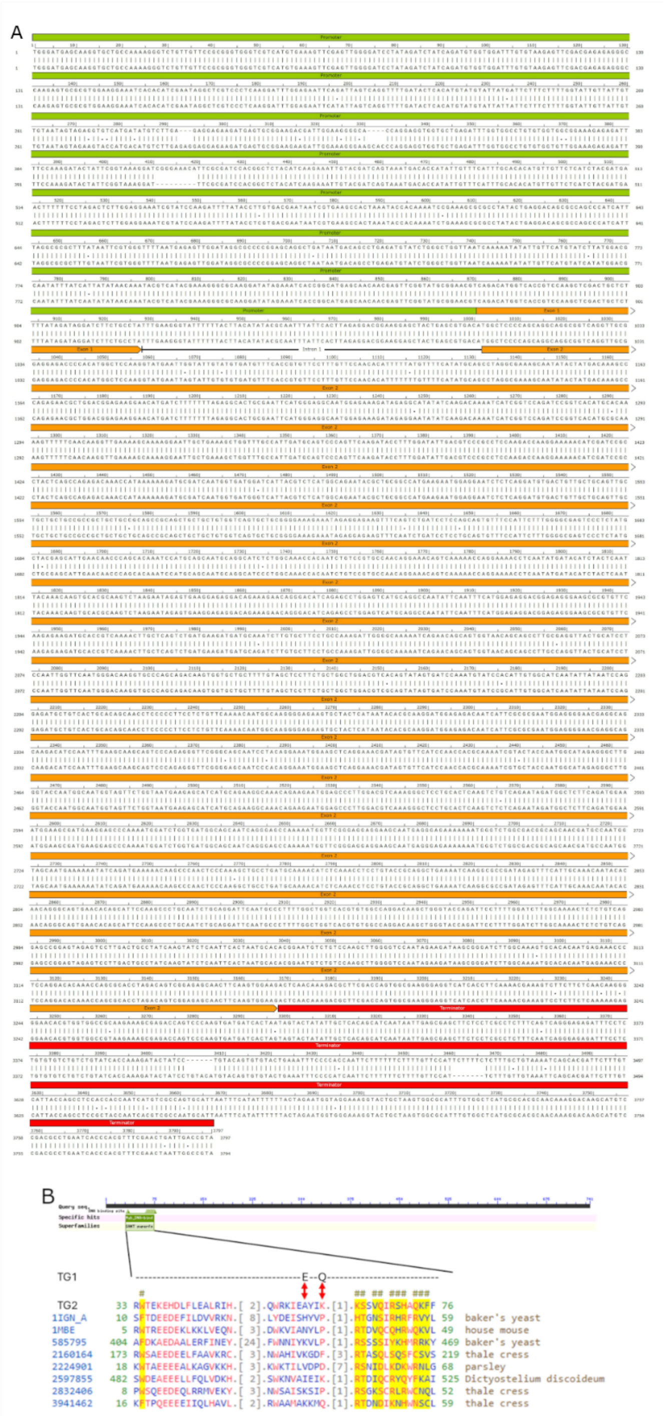

Supplementary Figure 2. *P. celeri* TG2 and TG1 CCA1 gene sequence comparison. (A) Pairwise alignment of the CCA1 genes from the two strains are displayed with 1 kb upstream promoter and 500 bp downstream terminator regions annotated on the upper track. The upper track displays the TG2 reference sequences with the TG1 on the lower track. (B) SANT/Myb-like DNA binding domain analysis and amino acid sequence alignments are displayed against the closest reference protein sequences with respect to TG2 CCA1 protein. The residue changes arising from non-synonymous SNPs in the TG1 genome are indicated above the alignment.

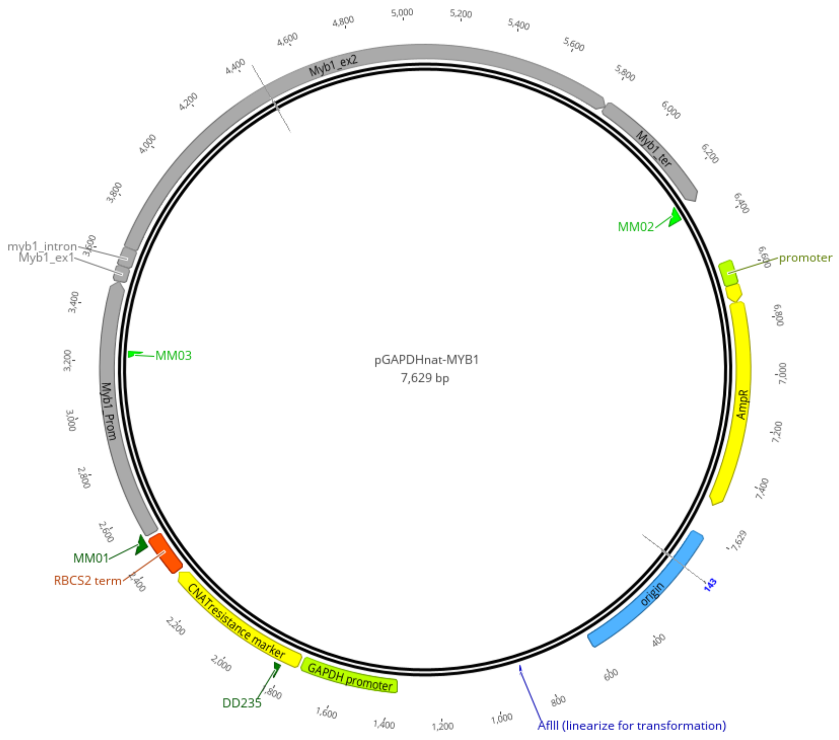

Supplementary Figure 3. Expression vector plasmid map of pGAPDHnat-MYB1.

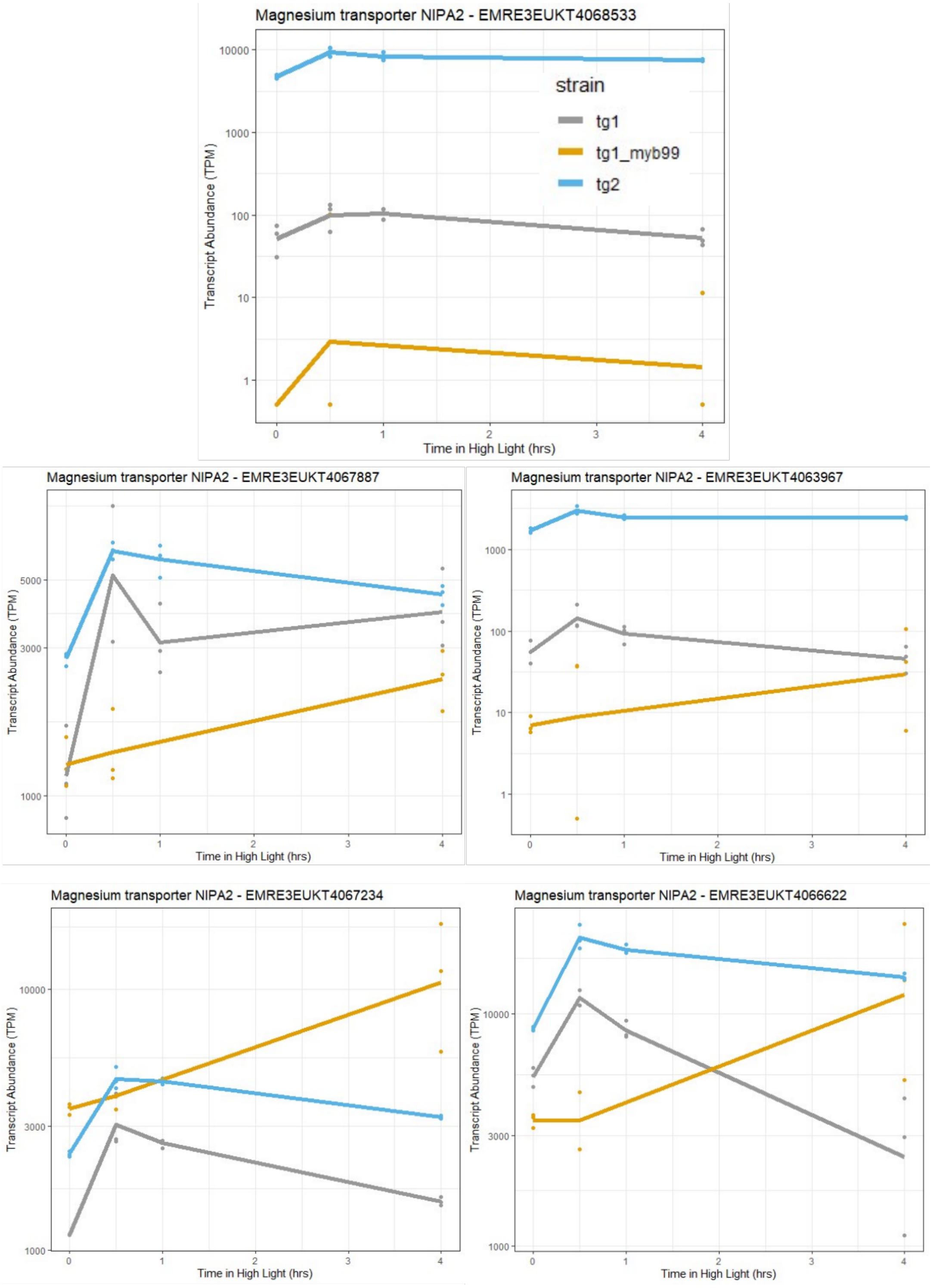

Supplementary Figure 4. *Picochlorum celeri* strains putative TG1-MYB99 transgene insertion site genes. Relative transcript levels for each biological replicate (n = 3) are displayed and colored according to strain. Values reported in transcripts per million mapped (TPM) following a regularized log transformation of the combined data set.

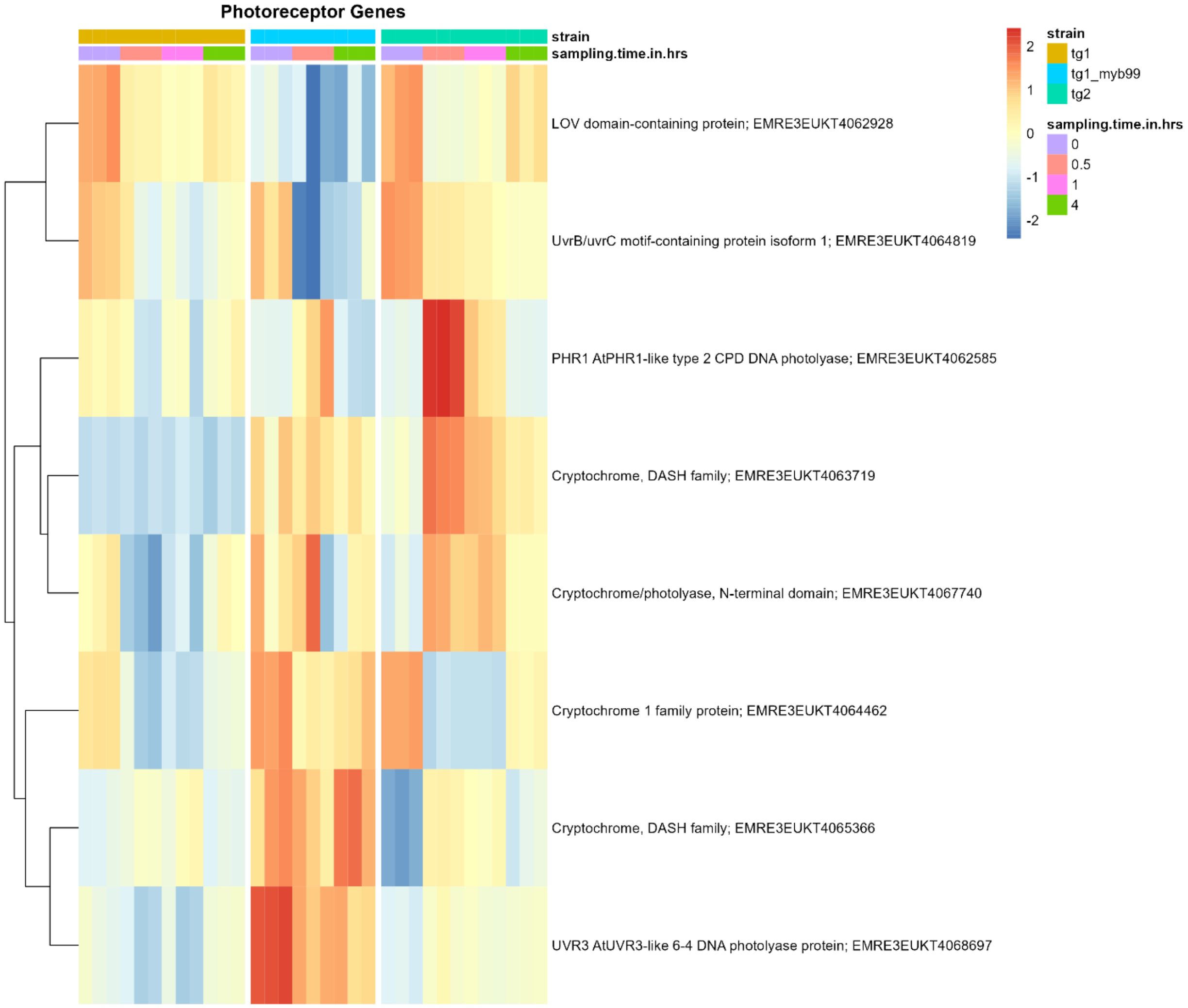

Supplementary Figure 5. *Picochlorum celeri* strains photoreceptor gene transcriptional response to HL growth. Data from each independent biological replicate (n = 3) are displayed with annotations for the strain and time in HL denoted by colors above columns. Colors represent transcript abundances derived from TPM values after scaling across rows to make genes comparable across samples.

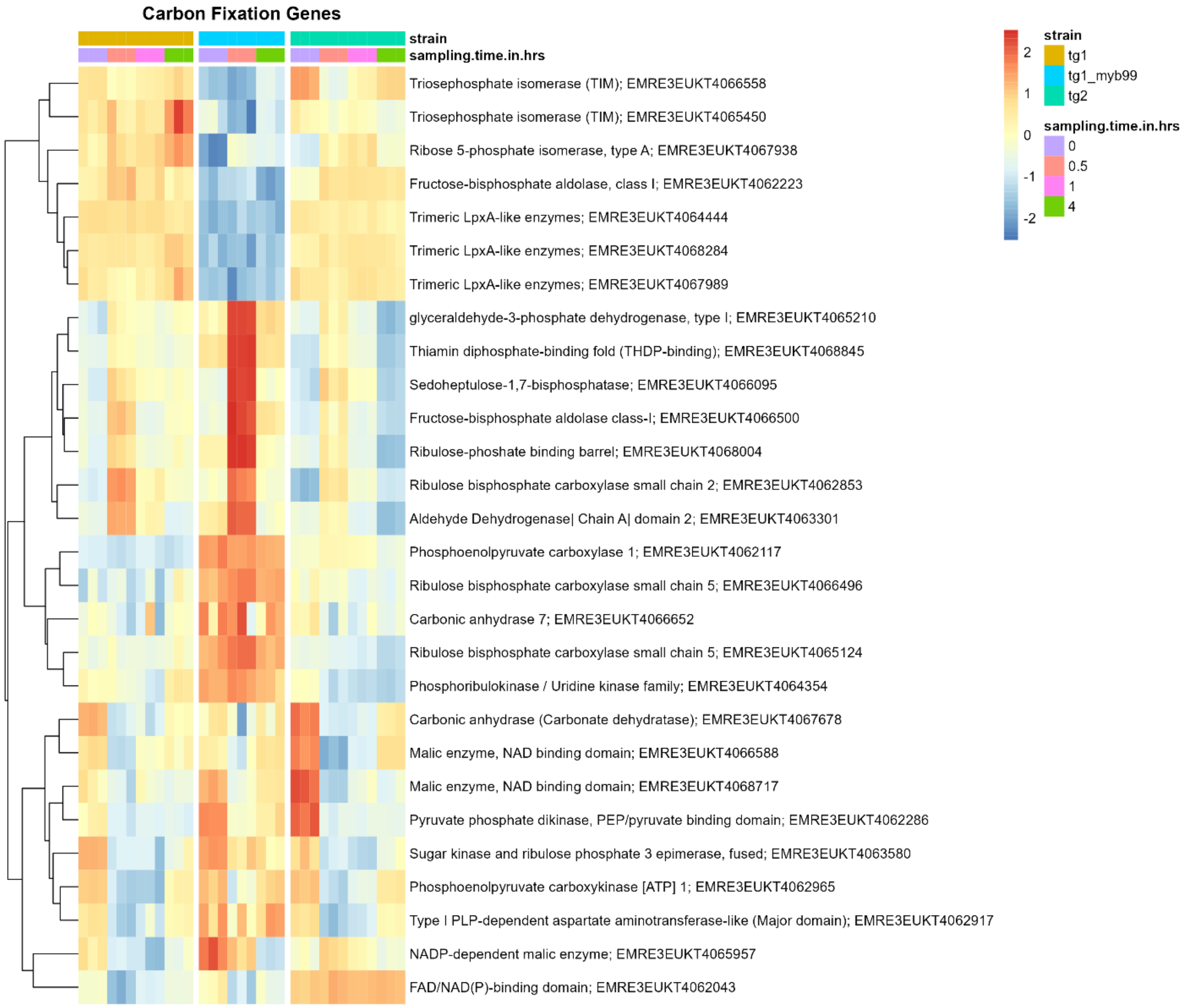

Supplementary Figure 6. *Picochlorum celeri* strains carbon fixation gene transcriptional response to HL growth. Data from each independent biological replicate (n = 3) are displayed with annotations for the strain and time in HL denoted by colors above columns. Colors represent transcript abundances derived from TPM values after scaling across rows to make genes comparable across samples.

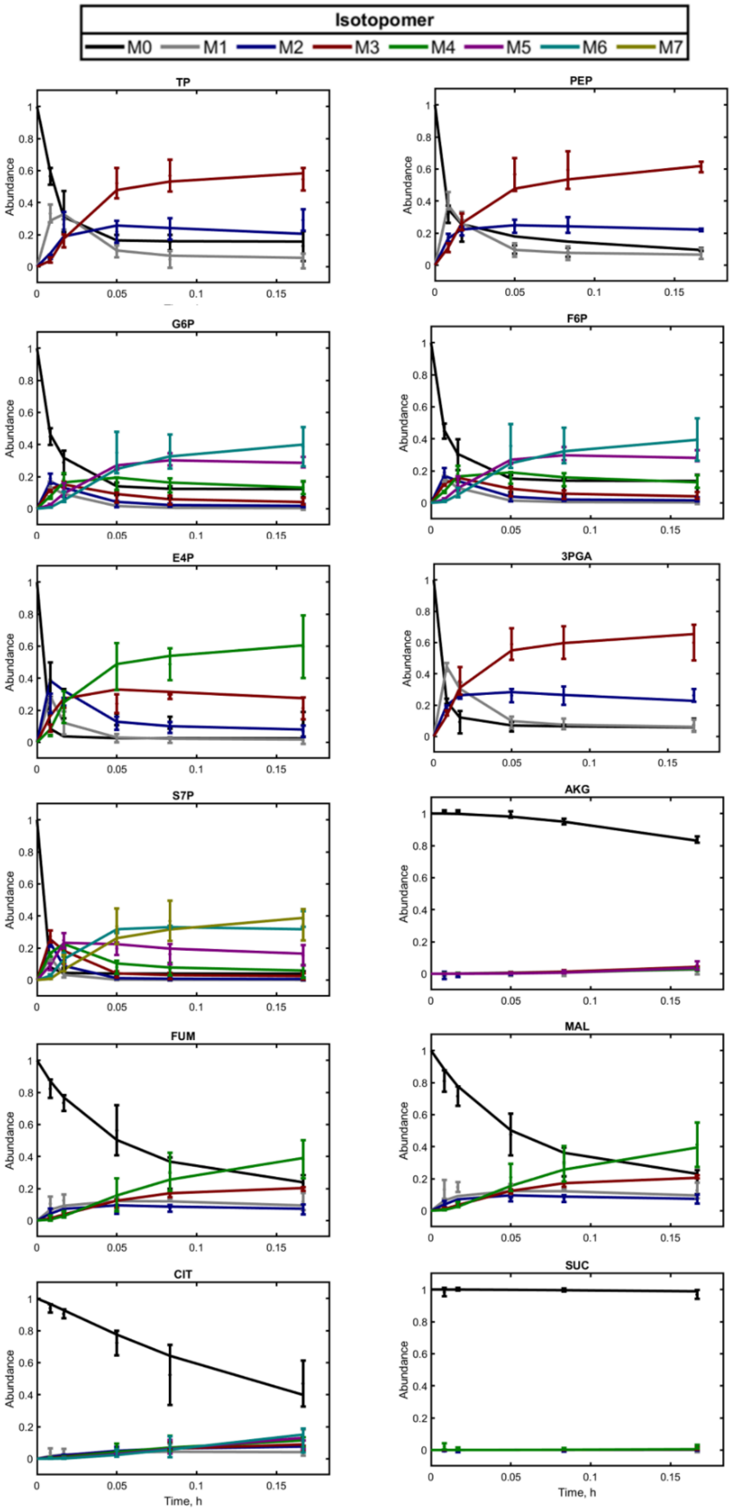

Supplementary Figure 7. Dynamic labeling of TG1. The dots represent the experimentally measured labeling pattern while the solid line represents the simulated fit using INCA. Experimental data is shown as mean ± SEM of three biological replicates (n = 3).

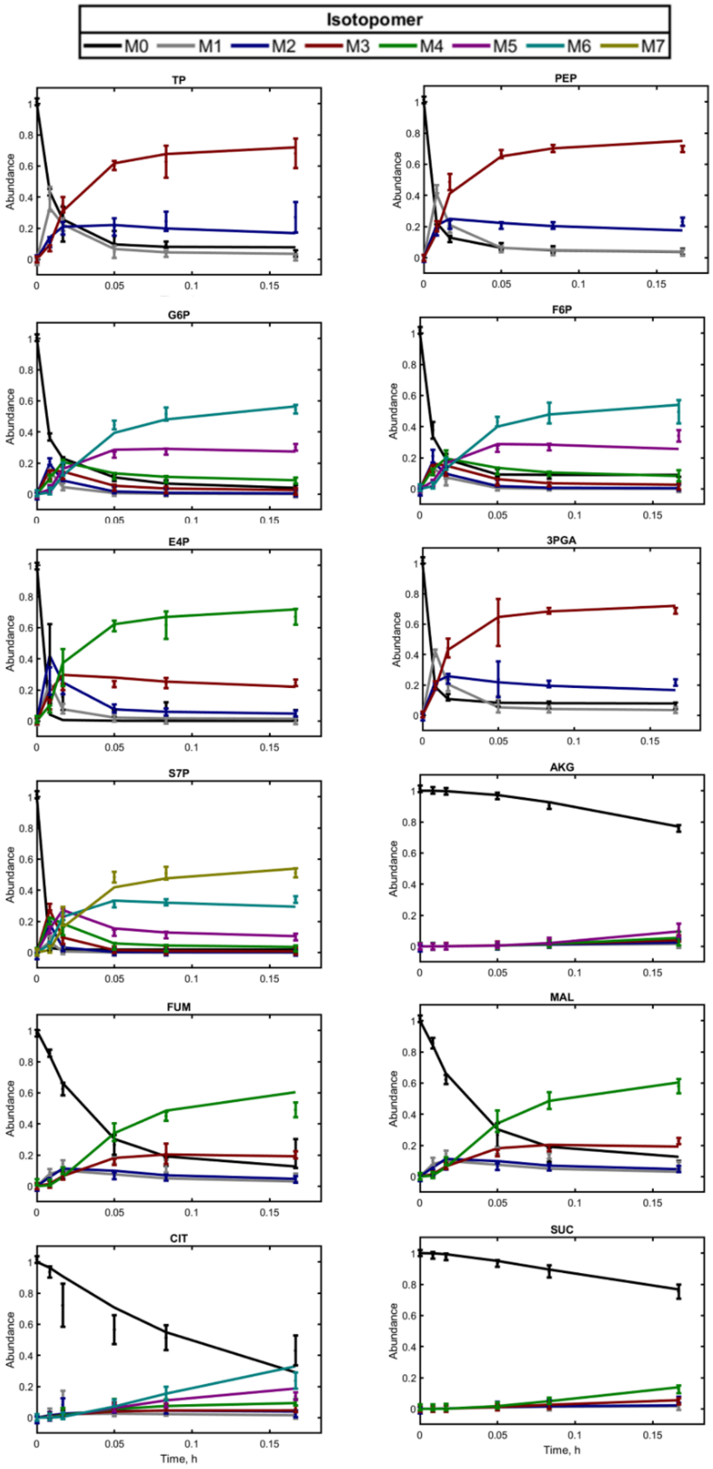

Supplementary Figure 8. Dynamic labeling of TG2. The dots represent the experimentally measured labeling pattern while the solid line represents the simulated fit using INCA. Experimental data is shown as mean ± SEM of three biological replicates (n = 3).

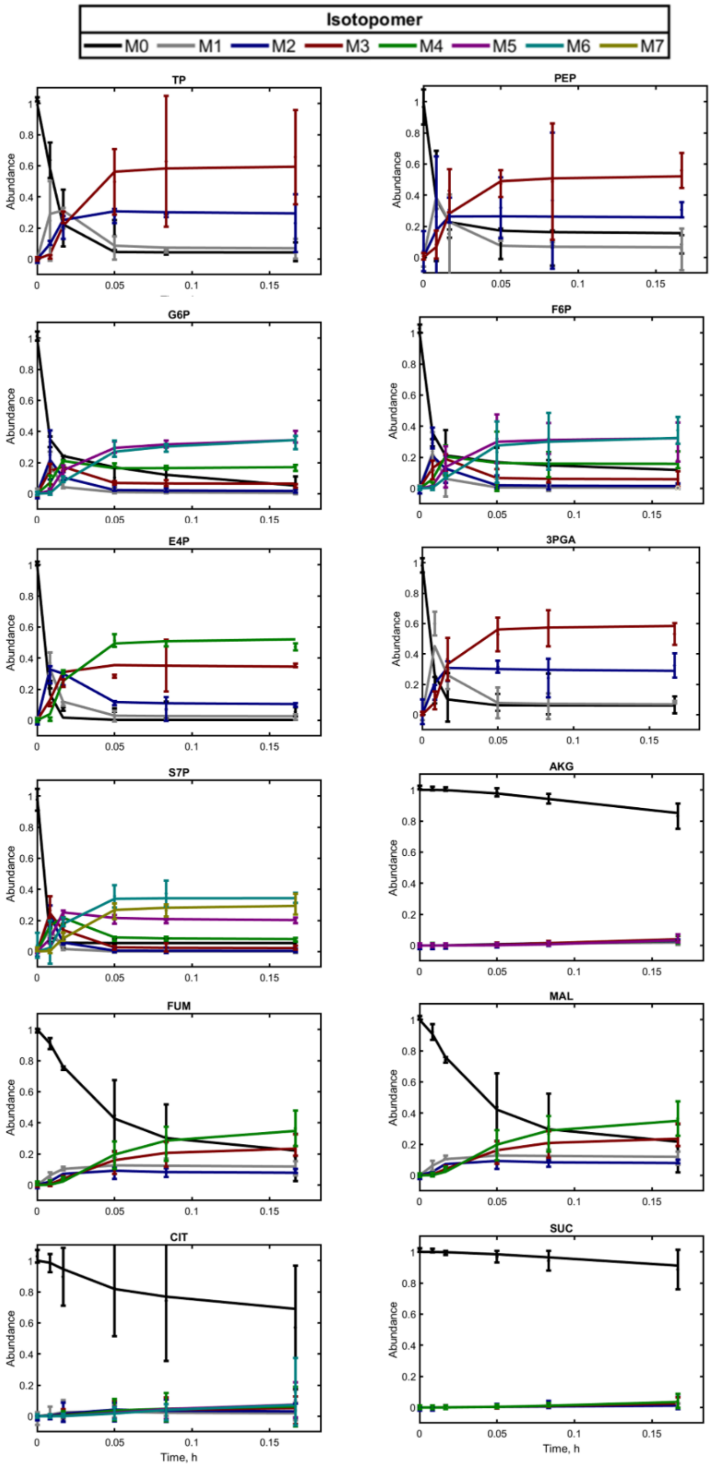

Supplementary Figure 9. Dynamic labeling of TG1-MYB99. The dots represent the experimentally measured labeling pattern while the solid line represents the simulated fit using INCA. Experimental data is shown as mean ± SEM of three biological replicates (n = 3).

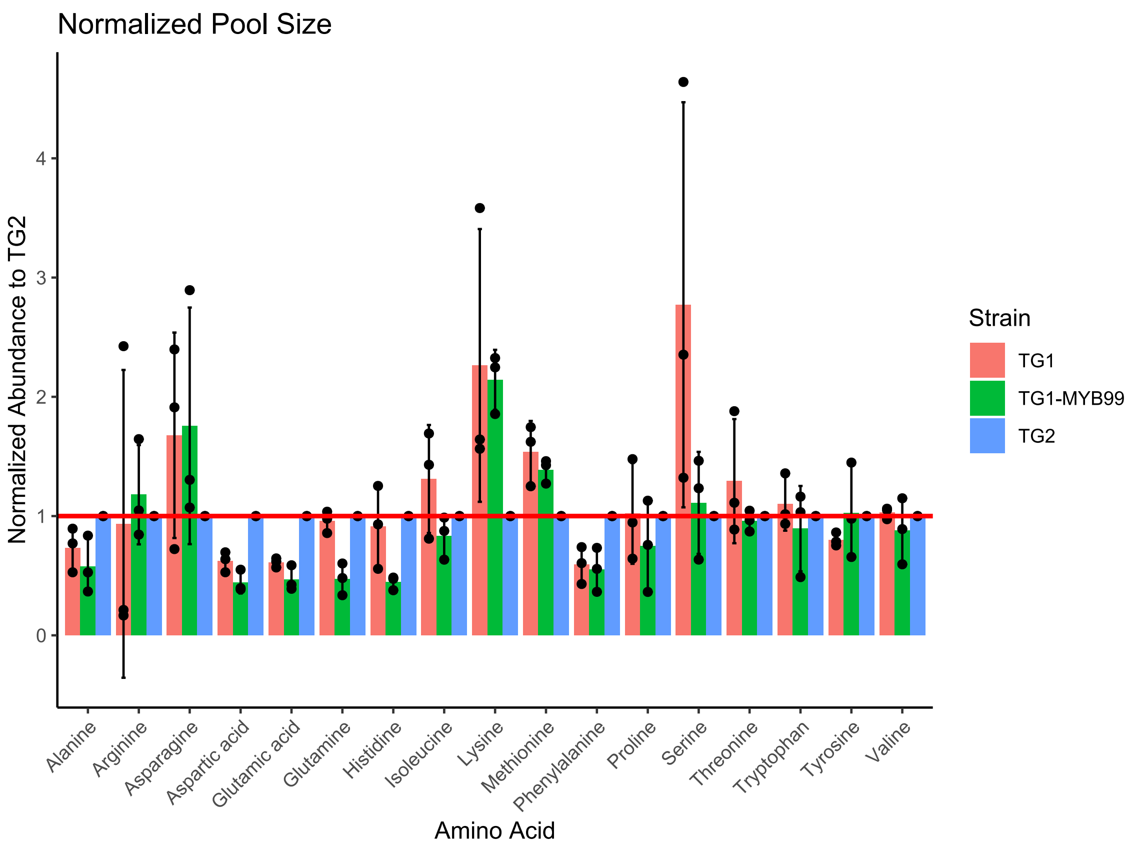

Supplementary Figure 10. Free amino acid pool size is normalized to TG2 level. The data represents the mean ± SD of three biological replicates (n = 3) utilized in the flux experiment. Quantities above and below the red line (y = 1) indicate pool size greater than or less than TG2 level.

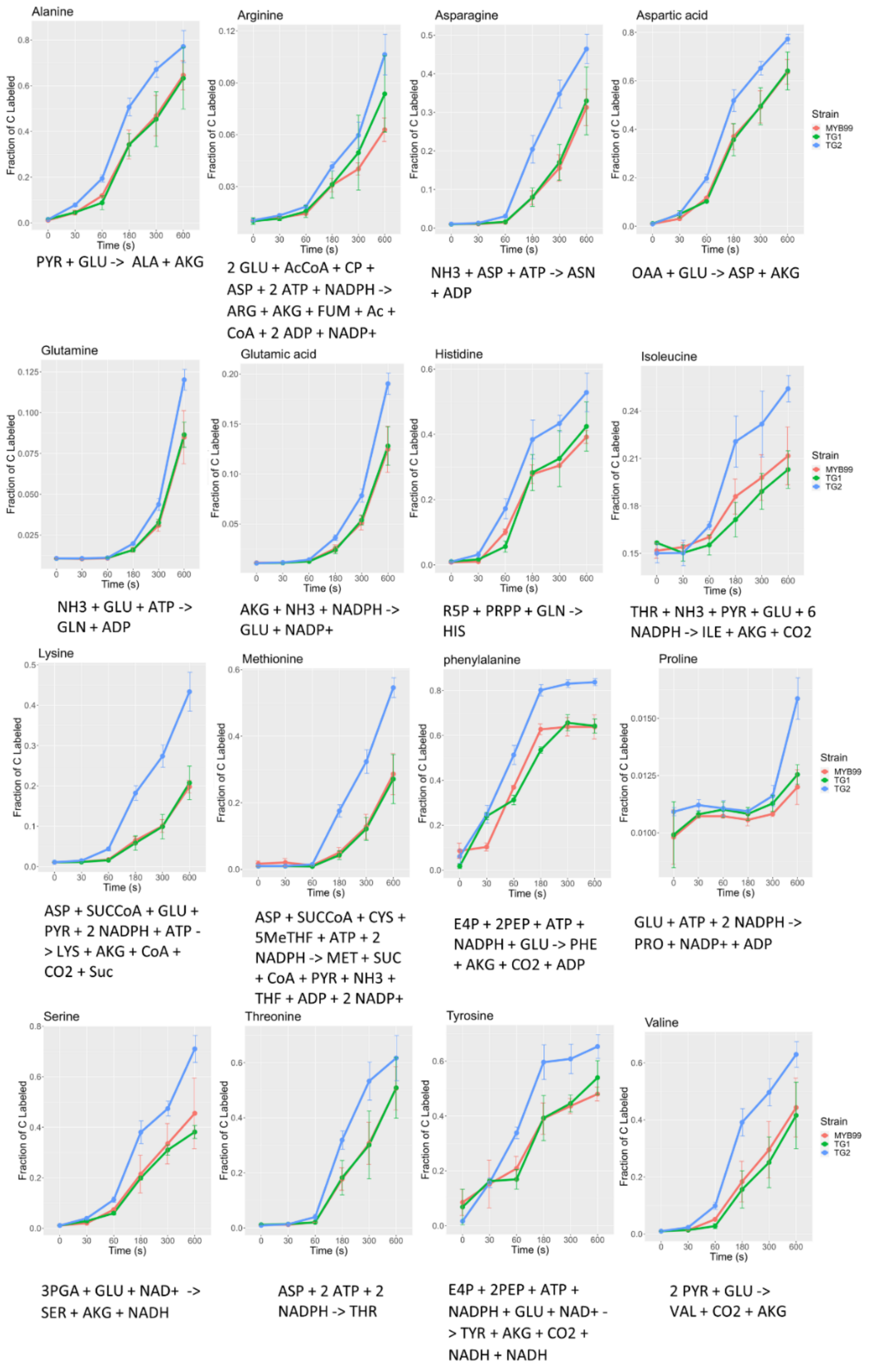

Supplementary Fig 11. Dynamic ^{13}C label incorporation in different amino acids. The fraction of C labeled is calculated as $\frac{1}{N} \cdot \sum_{i=1}^{N} M_i \cdot i$ where N is the total number of carbons in the metabolite, i is the label, and M_i is the fraction of ith isotopologue. The data represents mean ± SD of three biological replicates (n = 3). Metabolic reactions below each panel indicate the biosynthetic route of the amino acid that links it to the metabolites modeled in the flux model.

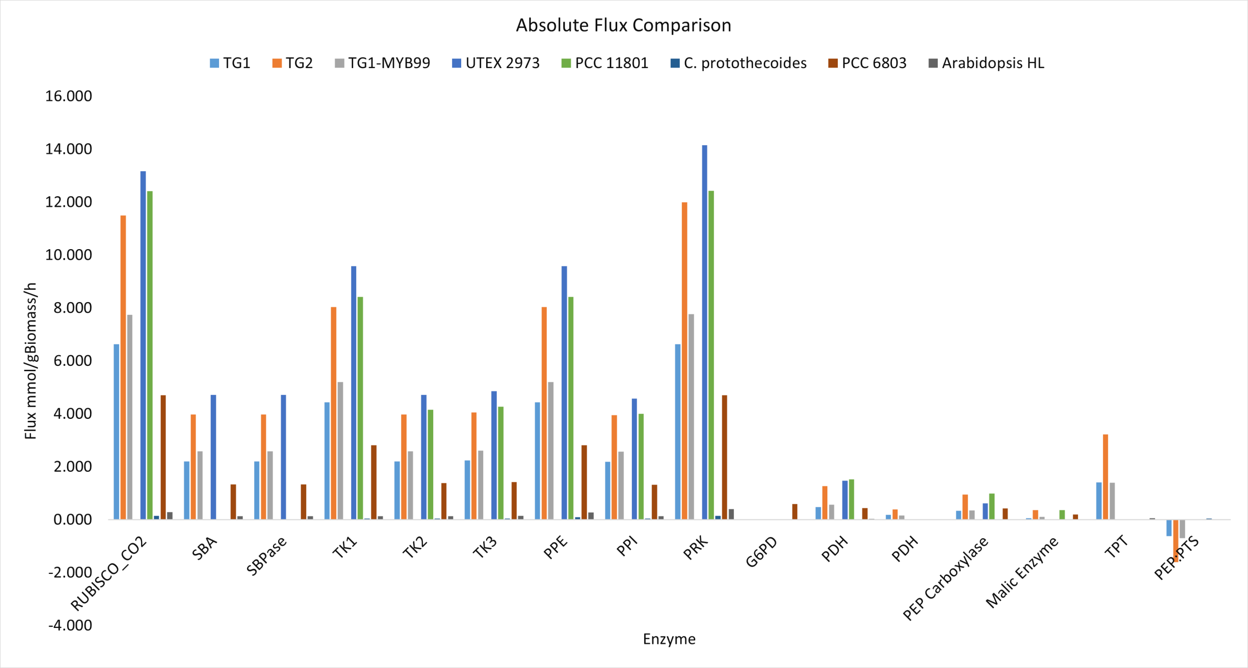

Supplementary Fig 12. Absolute flux of selected reactions from different organisms from metabolic flux analysis. TG1, TG2, and TG1-MYB99 were part of this study. Data for UTEX 2973[2], PCC 11801[8], *C. protothecoides*[4], PCC 6803[9], and Arabidopsis under high light[*10] were taken from literature. [*] Units are for mmol/gFW/h.

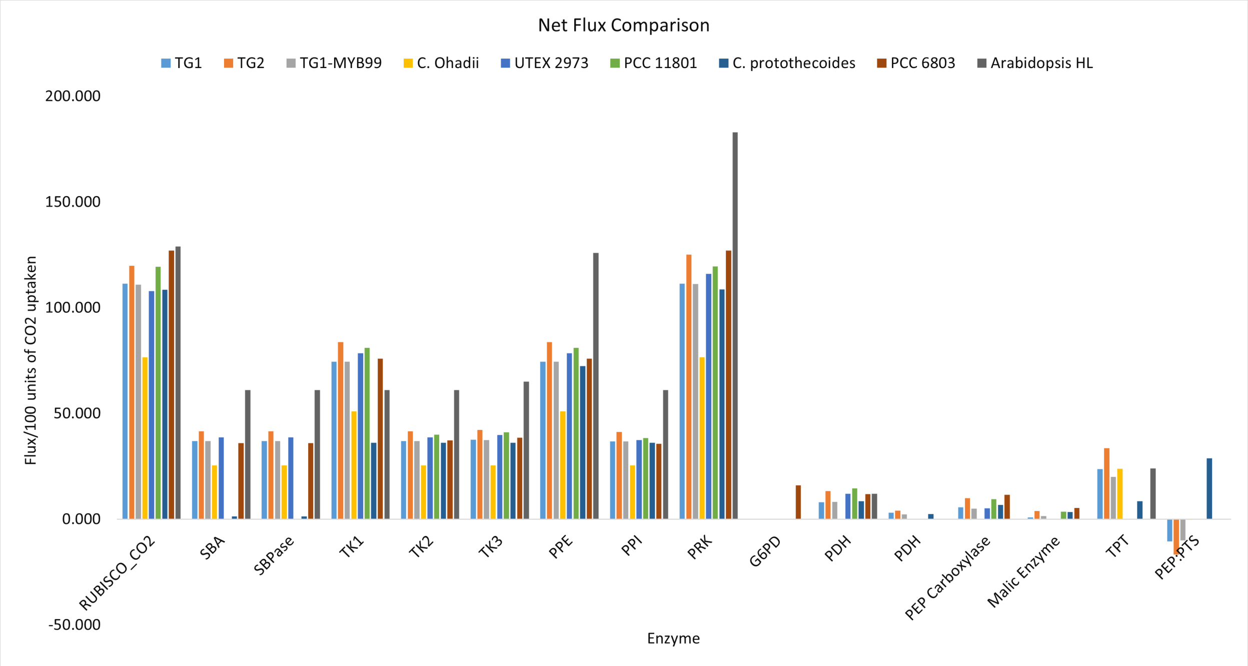

Supplementary Fig 13. Net flux of selected reactions from different organisms from metabolic flux analysis. Data were normalized for uptake of 100 units of CO_2. TG1, TG2, and TG1-MYB99 were part of this study. Data for UTEX 2973[2], PCC 11801[8], *C. protothecoides*[4], *C. ohadii*[3], PCC 6803[9], and Arabidopsis under high light[*10] were taken from literature. [*] Units are for mmol/gFW/h.

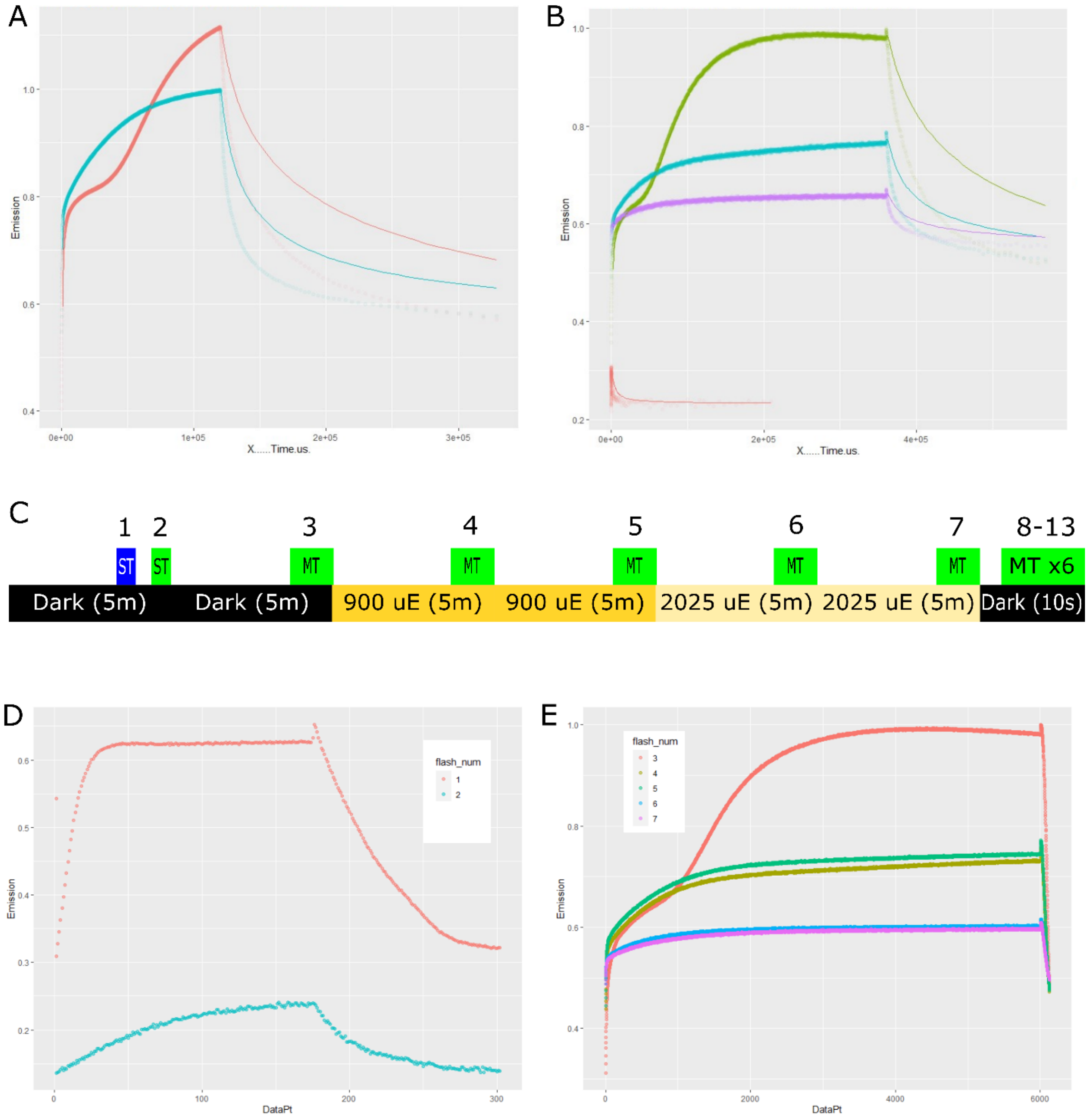

Supplementary Figure 14. Fast Repetition Rate fluorimetry custom method development. Preliminary experiments in *P. celeri* low density cell cultures did not reach full saturation using typical parameters for multiple turnover (MT) flash protocols (A). Subsequent increase in flashlet delay time and excitation light intensity resulted in fully saturated MT transients (B). Newly derived parameters were implemented in a combined protocol (C), that was used to determine Fv/Fm, sigma (D; flash 1,2), and Fm under increasing actinic light (E) for NPQ determination. Results displayed here are representative of method development data collected on *P. celeri* TG2-WT cells.

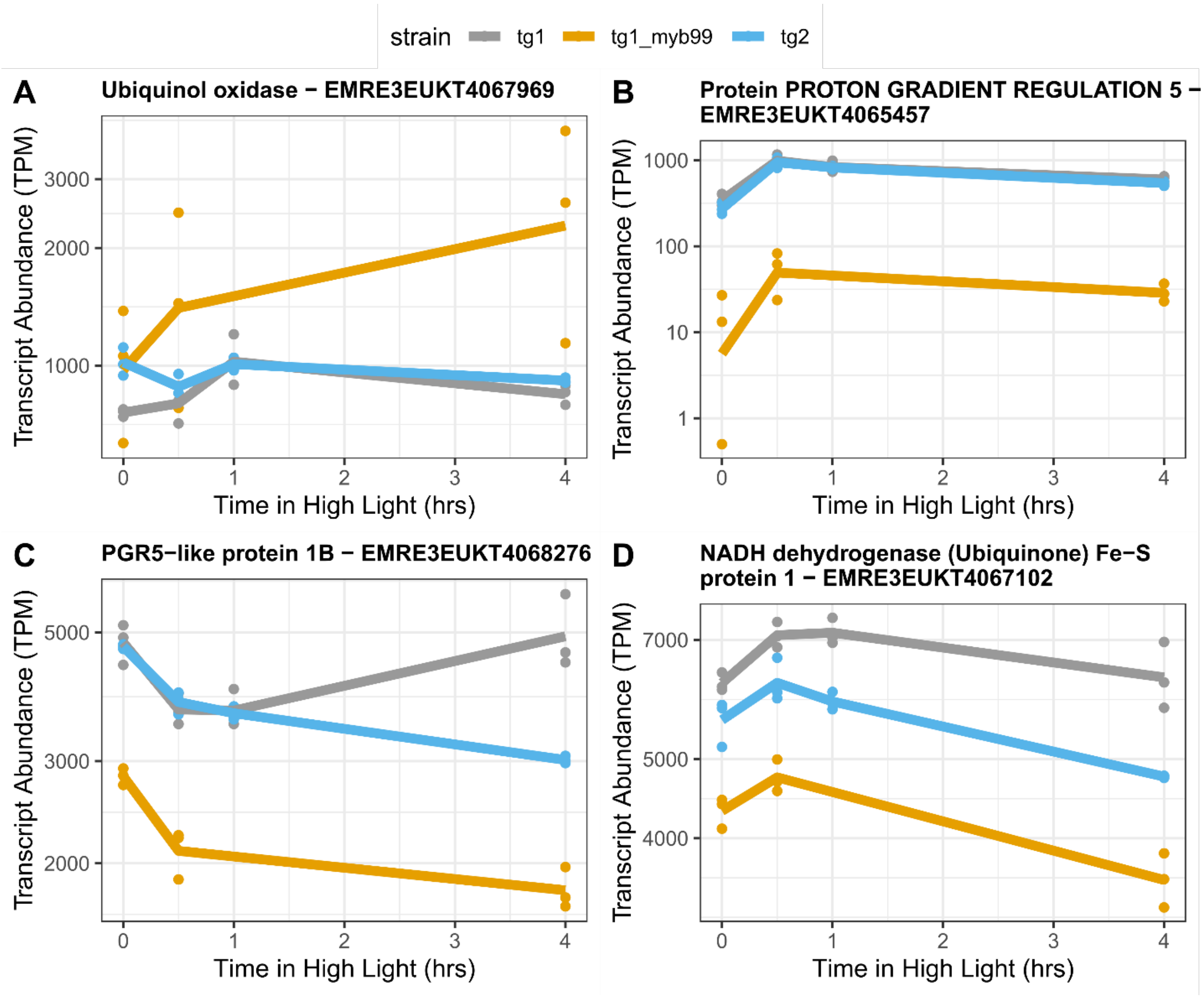

Supplementary Figure 15. Transcriptional response to HL growth for four selected alternative electron flow related genes; (A) Ubiquinol oxidase, (B) Protein Proton Gradient Regulation 5, (C) PGR5-like protein 1B, and (D) NADH dehydrogenase (Ubiquinone) Fe-S protein 1. Relative transcript levels for each biological replicate are displayed and colored according to strain. Values reported in transcripts per million mapped (TPM) following a regularized log transformation of the combined data set. Lines represent means of biological replicate data (n = 3).

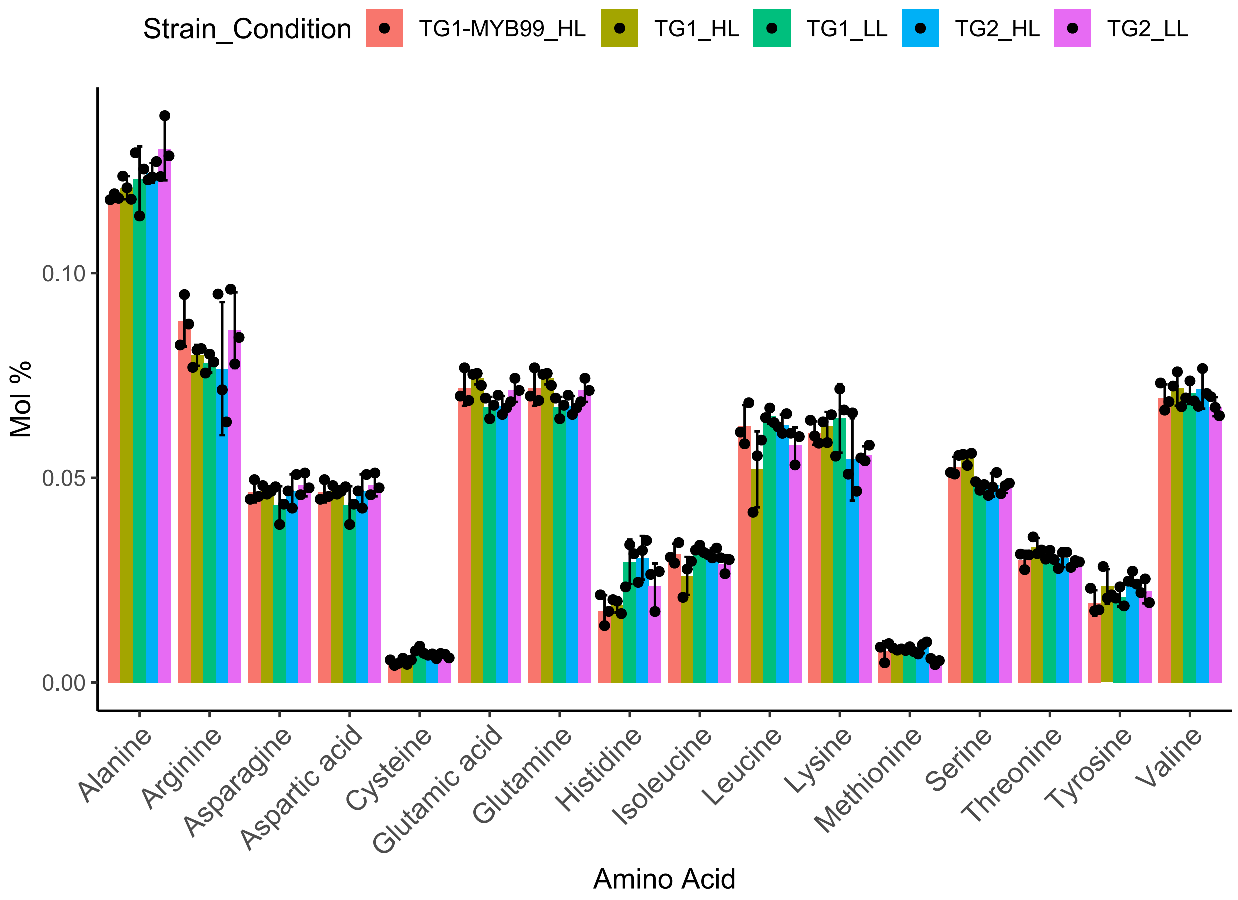

Supplementary Fig 16. Measured amino acid composition of hydrolyzed protein samples of TG1, TG2, and TG1-MYB99 which were grown under HL, and LL (See Methods: Growth Conditions). Data are presented as mean ± SD of three biological replicates (n = 3). Tryptophan is degraded in the sample preparation and thus is assumed to be that reported by aminoacids.com for TG2 (cultivated at HL to a high cell density). Phenylalanine, glycine, and proline do not have a reliable peaks and were assumed to be % reported by aminoacids.com for TG2. The assumed values for these amino acids are reported in **Supplementary Table 6.**

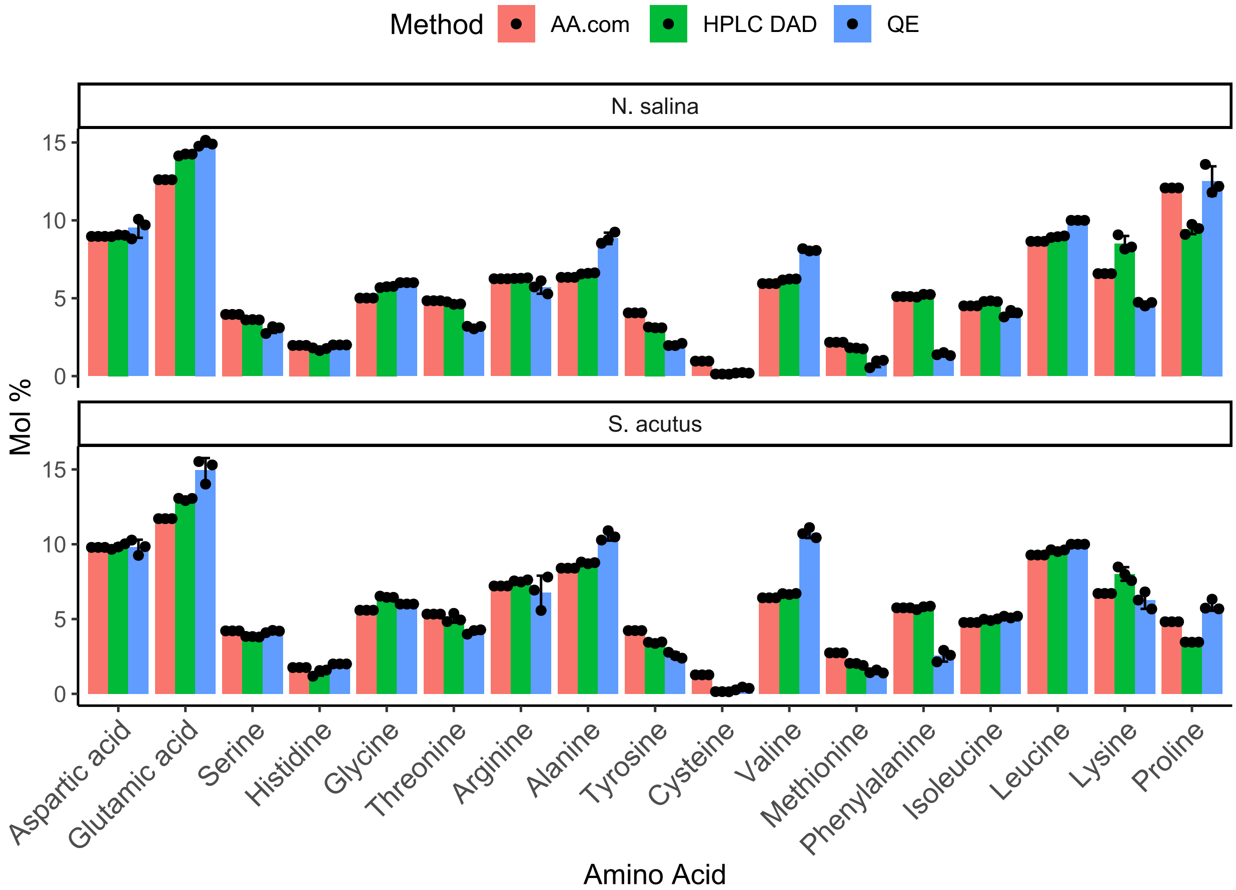

Supplementary Fig 17. Measured hydrolyzed protein amino acid composition with HILIC LC-MS method (QE), HPLC DAD, and aminoacids.com (AA.com) in *Nanochloropsis salina* and *Scenedesmus acutus*. Data are presented as mean ± SD of three technical replicates (n = 3).

Supplemental Discussion

Analysis of the TFBS upstream of the 149 genes sharing similar expression patterns to PGM found a lack of enrichment for the motifs most likely to match *P. celeri* CCA1 recognition sites, with LHY, CCA1, and RVE8 binding site p-values of 0.139 for each (**Supplementary Data 1**). This provides additional evidence for indirect regulation of starch synthesis genes. Over-represented TFBSs among the PGM cluster provide potential engineering targets and indicate likely connections to the wider regulatory network that was perturbed in the TG1-MYB99 mutant line. For instance, the NLP7 TFBS was highly enriched among this set and is documented to control a variety of plant nitrogen metabolism responses[11].

Prior fluxomics work in fast growing microalgae such as *C. ohadii*[3], *S. elongatus* UTEX 2973[2], and *S. elongatus* PCC 11801[8] and *C. protothecoides*[4], *Synechocystis* PCC 6803[9], and the plant *A. thaliana* (under high light)[10] allows for comparison with current *P. celeri* results. On an absolute flux scale, TG2 has a similar flux through RuBiscCO carboxylase activity compared to UTEX 2973, and PCC 11801 which have been characterized to have a comparable growth rate to TG2 (**Supplementary Fig 12**). When normalized for an uptake of 100 units of CO_2, we can compare organisms with vastly different growth rates. On this basis, *A. thaliana* shows the highest flux through several CBB enzymes (**Supplementary Fig 13**). This is particularly interesting since plants typically grow much slower compared to algae and cyanobacteria. One reason for this is that while *A. thaliana* shows a significant RuBisCO oxygenase activity which is not observed in any algae or cyanobacteria other PCC 6803.

References

1. Weissman, J. C. *et al.* High-light selection produces a fast-growing Picochlorum celeri. *Algal Research* **36**, 17–28 (2018).
2. Abernathy, M. H. *et al.* Deciphering cyanobacterial phenotypes for fast photoautotrophic growth via isotopically nonstationary metabolic flux analysis. *Biotechnology for Biofuels* **10**, 273 (2017).
3. Treves, H. *et al.* Carbon flux through photosynthesis and central carbon metabolism show distinct patterns between algae, C3 and C4 plants. *Nature Plants* **8**, 78–91 (2022).
4. Wu, C., Xiong, W., Dai, J. & Wu, Q. Genome-based metabolic mapping and 13C flux analysis reveal systematic properties of an oleaginous microalga Chlorella protothecoides. *Plant Physiol* **167**, 586–599 (2015).
5. Shastri, A. A. & Morgan, J. A. Flux Balance Analysis of Photoautotrophic Metabolism. *Biotechnology Progress* **21**, 1617–1626 (2005).
6. Kolber, Z. S., Prášil, O. & Falkowski, P. G. Measurements of variable chlorophyll fluorescence using fast repetition rate techniques: defining methodology and experimental protocols. *Biochimica et Biophysica Acta (BBA) - Bioenergetics* **1367**, 88–106 (1998).
7. Gorbunov, M. Y. & Falkowski, P. G. Using chlorophyll fluorescence kinetics to determine photosynthesis in aquatic ecosystems. *Limnology and Oceanography* **66**, 1–13 (2021).
8. Jaiswal, D., Nenwani, M. & Wangikar, P. P. Isotopically non-stationary ^{13}C metabolic flux analysis of two closely related fast-growing cyanobacteria, *Synechococcus elongatus* PCC 11801 and 11802. *The Plant Journal* tpj.16316 (2023) doi:10.1111/tpj.16316.

9. Young, J. D., Shastri, A. A., Stephanopoulos, G. & Morgan, J. A. Mapping photoautotrophic metabolism with isotopically nonstationary 13C flux analysis. *Metabolic Engineering* **13**, 656–665 (2011).
10. Ma, F., Jazmin, L. J., Young, J. D. & Allen, D. K. Isotopically nonstationary 13C flux analysis of changes in Arabidopsis thaliana leaf metabolism due to high light acclimation. *Proceedings of the National Academy of Sciences* **111**, 16967–16972 (2014).
11. Wang, H. *et al.* Regulatory functions of cellular energy sensor SnRK1 for nitrate signalling through NLP7 repression. *Nat. Plants* **8**, 1094–1107 (2022).
