# Supplementary material for: Central transcriptional regulator controls photosynthetic growth and carbon storage in response to high light
Source: Nat Commun. 2024 Jun 6;15:4842. doi: 10.1038/s41467-024-49090-7 (PMC11156908; doi:10.1038/s41467-024-49090-7)
Supplement: Supplementary file 3 — Description of Additional Supplementary Files [file 41467_2024_49090_MOESM3_ESM.pdf]

Description of Additional Supplementary Files

Supplementary Data Legends:

Supplementary Data 1: Enrichment analysis of identified transcription factors in clusters 4 and 21

Supplementary Data 2: Experimentally measured dynamic isotopomer distribution labeling patterns

Supplementary Data 3: Complete set of reactions and atom transitions in *P. celeri* metabolic network

Supplementary Data 4: Normalized net flux data with confidence intervals from INST-MFA

Supplementary Data 5: ATP and NADPH requirements, and % AEF (Alternative Electron Flow) estimated using metabolic fluxes from INST-MFA
