# Supplementary material for: Central transcriptional regulator controls photosynthetic growth and carbon storage in response to high light
Source: Nat Commun. 2024 Jun 6;15:4842. doi: 10.1038/s41467-024-49090-7 (PMC11156908; doi:10.1038/s41467-024-49090-7)
Supplement: Supplementary file 9 — Reporting Summary [file 41467_2024_49090_MOESM9_ESM.pdf]

nature portfolio

Corresponding author(s): Laurens, Lieve M.L.

Last updated by author(s): May 3, 2024

Reporting Summary

Nature Portfolio wishes to improve the reproducibility of the work that we publish. This form provides structure for consistency and transparency in reporting. For further information on Nature Portfolio policies, see our Editorial Policies and the Editorial Policy Checklist.

Statistics

For all statistical analyses, confirm that the following items are present in the figure legend, table legend, main text, or Methods section.

n/a	Confirmed	
☐	☒	The exact sample size (*n*) for each experimental group/condition, given as a discrete number and unit of measurement
☐	☒	A statement on whether measurements were taken from distinct samples or whether the same sample was measured repeatedly
☐	☒	The statistical test(s) used AND whether they are one- or two-sided<br>*Only common tests should be described solely by name; describe more complex techniques in the Methods section.*
☐	☒	A description of all covariates tested
☐	☒	A description of any assumptions or corrections, such as tests of normality and adjustment for multiple comparisons
☐	☒	A full description of the statistical parameters including central tendency (e.g. means) or other basic estimates (e.g. regression coefficient) AND variation (e.g. standard deviation) or associated estimates of uncertainty (e.g. confidence intervals)
☐	☒	For null hypothesis testing, the test statistic (e.g. F, t, r) with confidence intervals, effect sizes, degrees of freedom and P value noted<br>*Give P values as exact values whenever suitable.*
☒	☐	For Bayesian analysis, information on the choice of priors and Markov chain Monte Carlo settings
☒	☐	For hierarchical and complex designs, identification of the appropriate level for tests and full reporting of outcomes
☒	☐	Estimates of effect sizes (e.g. Cohen's *d*, Pearson's *r*), indicating how they were calculated

Our web collection on statistics for biologists contains articles on many of the points above.

Software and code

Policy information about availability of computer code

Data collection	RNAseq and whole genome data were collected by a commercial sequencing provider (Azenta, www.azenta.com using the Illumina platform)<br>Physiological data was collected using standard laboratory equipment, photophysiological analysis was performed with Fast Repetition Rate Fluorometer using standard software suite (https://soliense.com/index.php)<br>Metabolomics data was collected on a ThermoScientific QExactive Orbitrap mass spectrometer, with standard data collection, processing, and analysis software, Xcalibur v. 4.4. Peak integration was performed with TraceFinder 5.1.
Data analysis	No custom algorithms were created, all analyses followed assembled scripts with common RNAseq analysis code, including applications of the GSNAP, StringTie, samtools, Ballgown code in the R statistical analysis environment (representative examples of the code base can be found on a public repository on GitHub: https://github.com/ssteiche/pico_rnaseq), for metabolomics analysis MatLab R2022a was used, and INCA v2.2 computational platform (isotopomer network compartmental analysis) was run to solve the non-stationary metabolic flux results.

For manuscripts utilizing custom algorithms or software that are central to the research but not yet described in published literature, software must be made available to editors and reviewers. We strongly encourage code deposition in a community repository (e.g. GitHub). See the Nature Portfolio guidelines for submitting code & software for further information.

Data

Policy information about [availability of data]

All manuscripts must include a [data availability statement]. This statement should provide the following information, where applicable:

- Accession codes, unique identifiers, or web links for publicly available datasets
- A description of any restrictions on data availability
- For clinical datasets or third party data, please ensure that the statement adheres to our [policy]

The transcriptomic data generated in this study have been deposited in the NCBI SRA database under accession code PRJNA1024918 [https://www.ncbi.nlm.nih.gov/bioproject/?term=PRJNA1024918]. The genome assembly data used in this study are available in the NCBI database under accession code JAACMV000000000 [https://www.ncbi.nlm.nih.gov/nuccore/1811192891]. The metabolomics data generated in this study have been deposited in the Metabolomics Workbench database under accession code ST003035 [http://dx.doi.org/10.21228/M8P728]. The data shown in Supplementary_Data_4.xlsx was used to make Figure 3. All remaining source data used to make figures (Figures 3 and 4). are included in a separate source-data file, the source data for supplementary figures and analysis code is available upon request, with no conditions required to access the data and scripts aside from contacting the authors.

Research involving human participants, their data, or biological material

Policy information about studies with human participants or human data. See also policy information about sex, gender (identity/presentation), and sexual orientation and race, ethnicity and racism.

Reporting on sex and gender	NA
Reporting on race, ethnicity, or other socially relevant groupings	NA
Population characteristics	NA
Recruitment	NA
Ethics oversight	NA

Note that full information on the approval of the study protocol must also be provided in the manuscript.

Field-specific reporting

Please select the one below that is the best fit for your research. If you are not sure, read the appropriate sections before making your selection.

☒ Life sciences ☐ Behavioural & social sciences ☐ Ecological, evolutionary & environmental sciences

For a reference copy of the document with all sections, see nature.com/documents/nr-reporting-summary-flat.pdf

Life sciences study design

All studies must disclose on these points even when the disclosure is negative.

Sample size	We have previously determined that triplicate biological cultures in the simulated algae growth environment reactor that are sufficient based on experience to give us a representation of physiology repeatability within a 95% confidence interval. N=3 was chosen for 13C labeling experiments to provide adequate replication and avoid excessive 13C label costs. Transcriptomic experiments were conducted on independent biological replicate cultures (N=3) for each of the two wild type strains (TG1 and TG2) and the mutant TG1-MYB99 cell line. Re-sampling of each replicate culture was utilized to collect time course samples for each point described in the manuscript.
Data exclusions	None of the biological replicates were excluded for any of the reported results, including the 13C labeling experiments or for the transcriptomic experiments. All sampled data were analyzed and reported in the manuscript with associated statistical significance testing.
Replication	Reproducibility was verified based on presented and many prior experiments, including experience in our laboratory on biological and physiological repeatability in cultivation reactors, by using N=3 biological replicates for all experiments. The observed close repeatability as illustrated in the principal component analysis of the RNAseq data supports our selection of N=3 for sequencing and illustrates that time point sampling captured the respective strain and time variables. All variation and replication is reported in the manuscript alongside the results.
Randomization	Metabolomics and fluxomics time point samples were collected from biological reactors with N=3 replication and analyzed by LC-MS in a randomized order. Cultures for transcriptomics experiments were distributed in a non-random alternating positional design in the controlled environment reactor, to account for minor edge co-variation in light irradiance from LED panels.
Blinding	The same researcher performed 13C labeling, sample prep, and LC-MS data collection and thus there was no blinding, however the randomized sequence of LC queuing and automation of the data collection, peak identification and analysis pipelines minimized researcher-

induced bias. Blinding was not applicable for transcriptomics experimental design (one researcher collected the samples), the sequencing was carried out by a commercial sequencing company (Azenta).

Reporting for specific materials, systems and methods

We require information from authors about some types of materials, experimental systems and methods used in many studies. Here, indicate whether each material, system or method listed is relevant to your study. If you are not sure if a list item applies to your research, read the appropriate section before selecting a response.

Materials & experimental systems

n/a	Involved in the study	
☒	☐	Antibodies
☒	☐	Eukaryotic cell lines
☒	☐	Palaeontology and archaeology
☒	☐	Animals and other organisms
☒	☐	Clinical data
☐	☒	Dual use research of concern
☐	☒	Plants

Methods

n/a	Involved in the study	
☒	☐	ChIP-seq
☒	☐	Flow cytometry
☒	☐	MRI-based neuroimaging

Dual use research of concern

Policy information about dual use research of concern

Hazards

Could the accidental, deliberate or reckless misuse of agents or technologies generated in the work, or the application of information presented in the manuscript, pose a threat to:

No	Yes	
☒	☐	Public health
☒	☐	National security
☒	☐	Crops and/or livestock
☐	☒	Ecosystems
☒	☐	Any other significant area

Hazards

A genetically engineered microalgal cultivar was created in this work with a fitness advantage over the wild type original strain, current presented research does not include interactions with natural ecosystems. We are aware of TSCA (Toxic Substances Control Act) required approvals for any future outdoor testing with documentation of environmental release risk, that will include a complete biosecurity assessment and natural ecosystem risk analysis.

For examples of agents subject to oversight, see the United States Government Policy for Institutional Oversight of Life Sciences Dual Use Research of Concern.

Experiments of concern

Does the work involve any of these experiments of concern:

No	Yes	
☒	☐	Demonstrate how to render a vaccine ineffective
☒	☐	Confer resistance to therapeutically useful antibiotics or antiviral agents
☒	☐	Enhance the virulence of a pathogen or render a nonpathogen virulent
☒	☐	Increase transmissibility of a pathogen
☒	☐	Alter the host range of a pathogen
☒	☐	Enable evasion of diagnostic/detection modalities
☒	☐	Enable the weaponization of a biological agent or toxin
☒	☐	Any other potentially harmful combination of experiments and agents

Precautions and benefits

Biosecurity precautions

No specific precautions beyond the NREL instutional biosafety research guidance for working with genetically engineered organisms were necessary during the experimental work reported here, future applications of the findings will take biosecurity, release risk and mitigation, and approvals into account

Biosecurity oversight	NA
Benefits	The transcriptional regulator identified in this work has potential to be implicated in future crop yield improvement strategies, and the work in model algae is warranted as a rapid physiological test system
Communication benefits	Future research will determine the possible impact of this work

Plants

Seed stocks	This work is based on isolated and enriched wild type algae strains from the Texas Gulf Coast (TG1 and TG2), from prior published work by one of the co-authors (Dr. Weissman), original work is referenced throughout
Novel plant genotypes	One genetically engineered mutant line (TG1-MYB99) is reported with detailed description of the transformation procedures as detailed in the manuscript, with supplemental file including the vector design and in depth method descriptors.
Authentication	A full genome sequence was collected on the mutant line, allowing us to identify and localize the insertion location (as described in the manuscript) and can be made available.
